# Distillation Style Regulators and Semantic Prior-Guided Framework for Non-Ideal Single-View 3D Vehicle Point Cloud Reconstruction

**DOI:** 10.3390/s26113359

**Published:** 2026-05-26

**Authors:** Jinghao Cao, Xiajun Liu, Rui Xue

**Affiliations:** 1 School of Mechanical Engineering, Jiangsu University of Science and Technology, Zhenjiang 212100, China; 2 School of Electronic Science and Engineering, Nanjing University, Nanjing 210023, China; 3 School of Internet of Things Engineering, Jiangnan University, Wuxi 214122, China; 4 School of Energy and Power Engineering, Nanjing Institute of Technology, Nanjing 211167, China

**Keywords:** 3D reconstruction, semantic prior-guided learning, distillation-style regulators multimodal, information fusion

## Abstract

The closed-loop testing of autonomous driving systems critically depends on large-scale libraries of diverse and realistic 3D vehicle assets, yet current pipelines still rely on labor-intensive modeling or multi-view capture, making efficient construction a key bottleneck. To overcome this bottleneck and enable convenient, cost-effective 3D asset generation, we propose a semantic prior-guided framework for accurate and robust vehicle point cloud reconstruction from casually captured single-view photographs. Our framework is built on a diffusion backbone but is fundamentally driven by two forms of prior knowledge: First, geometric and appearance priors from camera-aware image features, masks, and distance-transform maps are projected onto the evolving point cloud, compensating for the severe information loss in single-view inputs. Second, we introduce distillation-style regulators—pretrained neural networks that encode vehicle type and model semantics; they act as teacher networks that impose high-level constraints on the generated point clouds, transferring rich semantic knowledge and effectively regularizing the learning process. With these priors, our model infers vehicle-specific semantics from limited observations and reconstructs high-quality 3D point cloud assets. On the 3DRealCar++ dataset, our method clearly surpasses state-of-the-art point cloud baselines in both F-score and Chamfer Distance.

## 1. Introduction

The construction of large-scale, diverse, and realistic 3D vehicle assets has become a central bottleneck for closed-loop testing of autonomous driving systems. While current research shows that the primary limitation of autonomous driving algorithms lies in data scarcity and the resulting poor handling of corner cases [1], collecting such rare and often dangerous scenarios (e.g., collisions, near-misses) in the real world is constrained by safety, ethical, and legal concerns [2,3]. Closed-loop testing addresses this issue by evaluating autonomous vehicles in virtual environments, enabling rapid, safe, and cost-effective iteration of algorithms [3]. However, the realism and coverage of these tests are fundamentally limited by the quality and diversity of the underlying 3D assets.

Early closed-loop testing mainly relied on simulation environments, where scenes were crafted using 3D rendering tools and computer graphics techniques [4,5,6]. Despite the visual fidelity of modern simulators, researchers soon identified a noticeable gap between results obtained in simulation and those in the real world [7]. This has led to a shift toward constructing 3D assets directly from real-world data via 3D reconstruction, and then using these assets for more faithful closed-loop evaluation [8]. To further reduce manual effort and cost, recent work explores reconstructing 3D vehicle assets from relatively simple and readily available inputs, such as single images or sparse views [9,10,11]. Despite recent progress in single-image 3D reconstruction, existing vehicle reconstruction frameworks still suffer from two major limitations: First, most existing methods primarily rely on visual observations while neglecting semantic prior information such as vehicle brand, model, and category attributes, which are highly correlated with global geometric structure. Second, general-purpose reconstruction frameworks often assume relatively complete or ideal observations and, therefore, struggle under real-world non-ideal conditions involving occlusion, truncation, arbitrary viewpoints, and incomplete structural information.

Therefore, we propose a novel perspective: **Can we design a reliable algorithm to reconstruct the 3D assets of vehicles with specific types and brands from casually taken images captured from non-ideal viewpoints?** Addressing and exploring this problem will constitute the primary focus of this work. Thanks to the rapid advances in techniques like Gaussian splatting [12] and NeRF [13], reconstructing a 3D scene or object from multiple images and their associated camera parameters has become significantly more accessible. In contrast, single-view 3D reconstruction is a highly ill-posed problem. Reconstructing 3D objects from a single image, where both geometric and semantic information are limited, has increasingly attracted attention in recent research.

In many practical closed-loop testing scenarios, simply representing vehicles as generic “cars” is no longer sufficient. Perception performance, interaction behavior, and safety margins depend strongly on fine-grained properties such as vehicle type (e.g., SUV, truck, sedan) and specific model (e.g., compact hatchback vs. long-wheelbase sedan). For example, accurate modeling of occlusions, visibility, and sensor returns depends on the true size and shape of the vehicle; realistic simulation of traffic flow and collision risk requires distinguishing between heavy trucks and small passenger cars; and scenario libraries for functional safety and regulation testing often specify particular brands and models involved in corner cases. Therefore, being able to reconstruct vehicles with specific types and brands is critical for building high-fidelity, semantically consistent 3D asset libraries [14,15].

**Figure 1:**
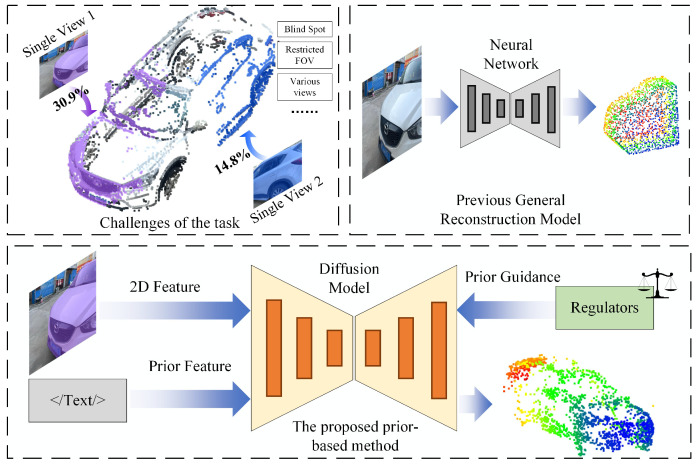
Motivation of the proposed prior-guided reconstruction framework. The **upper-left** image illustrates a casually captured vehicle image under non-ideal conditions, where severe viewpoint limitations, occlusions, truncation, and missing structural information introduce strong ambiguity into single-view reconstruction. The **upper-right** image shows that directly applying a general-purpose image-to-3D reconstruction framework to such inputs may lead to incomplete or semantically incorrect 3D assets. The **bottom** image illustrates that introducing semantic and geometric prior guidance during the generation process enables the model to better understand vehicle structure and generate more plausible and semantically consistent 3D point cloud assets.

This work focuses on the following key question: **Given a casually captured single image of a vehicle from a non-ideal viewpoint, can we reliably reconstruct a 3D point cloud asset that is both geometrically plausible and semantically faithful to its specific type and model?** This setting is highly challenging. Real-world images typically exhibit occlusions, truncated views, and cluttered backgrounds; single-view 3D reconstruction is inherently ill-posed due to severe depth and shape ambiguities; and existing methods are often trained on idealized datasets and treat all vehicles uniformly as “cars”, ignoring fine-grained semantics. As illustrated in Figure 1, vehicle images captured in real-world scenarios are often obtained from arbitrary viewpoints and under non-ideal conditions. Such images frequently contain severe occlusions, truncation, incomplete contours, and missing structural regions, making single-view reconstruction highly ambiguous. In these situations, the visible image regions are often insufficient for reliably inferring the complete global geometry of the vehicle.

When these non-ideal inputs are directly processed by general-purpose image-to-3D reconstruction frameworks, the generated results may become severely distorted or semantically inconsistent. This is because universal reconstruction models typically rely heavily on visible image observations and lack sufficient prior understanding of vehicle-specific geometric structures and semantic characteristics. As a result, they may reconstruct only partial structures or generate geometrically implausible assets that do not match the actual vehicle category or shape.

This observation reveals an important gap between generic reconstruction frameworks and practical autonomous-driving asset reconstruction scenarios. Therefore, we argue that introducing semantic and geometric prior information during the generation process is necessary for guiding the model toward structurally plausible and semantically consistent vehicle reconstruction under non-ideal single-view conditions.

Under these conditions, existing universal reconstruction frameworks tend to generate incomplete geometries, semantically inconsistent structures, or reconstructions that are biased toward the visible regions only. These failure cases reveal that purely image-driven reconstruction methods are insufficient when the input observations are sparse or partially missing. Therefore, introducing semantic and geometric prior knowledge becomes necessary for recovering structurally plausible and semantically consistent vehicle point clouds under non-ideal single-view scenarios..

To cope with these challenges, we choose 3D point clouds as the target representation. Compared with meshes, point clouds impose weaker topological constraints and are more tolerant to missing or uncertain regions, reducing the risk of severe geometric artifacts under non-ideal viewpoints. At the same time, point clouds serve as a flexible intermediate representation: given additional information or stronger downstream modules, they can be converted into meshes or textured surface models with high fidelity [16].

**Figure 2:**
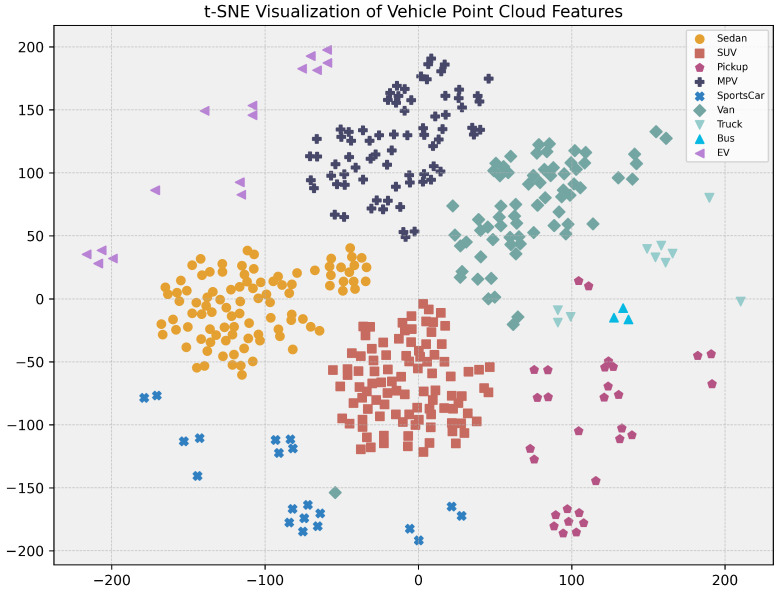
The visualized mapping of vehicle point cloud feature distributions categorized by different vehicle types. It can be observed that the point cloud features of different vehicle types exhibit clear correlations. Therefore, the vehicle type information is considered to be an important prior and can serve as a guiding condition.

**Figure 3:**
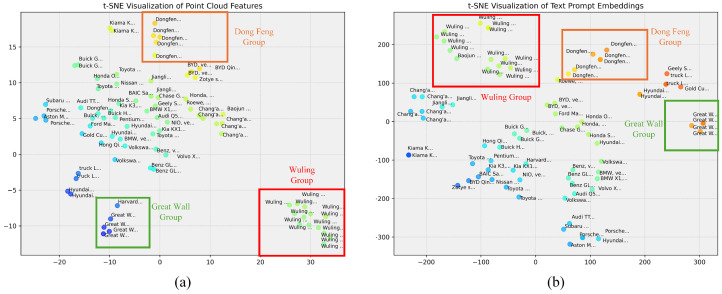
A t-SNE visualization of semantic distributions in different feature spaces. (**a**) Point cloud feature embeddings extracted by the proposed framework. (**b**) Corresponding text-prompt embeddings generated from semantic priors. Representative clusters corresponding to semantically related vehicle groups are highlighted for qualitative comparison. The visualization demonstrates that both point cloud features and text-prompt embeddings exhibit similar neighborhood distribution tendencies and semantic clustering structures, indicating effective semantic alignment between geometric representations and textual priors.

Beyond conceptual motivation, we also conduct a feasibility study to verify that vehicle type and textual semantics provide informative priors for point cloud reconstruction. Following feature [17] extraction from vehicle point clouds by our proposed Vehicle Type Regulator, we apply t-SNE [18] to visualize their distributions across different vehicle types; clear clusters emerge for categories such as “Sedan” and “SUV” (Figure 2), indicating that type labels correlate strongly with geometric structure and can serve as effective guidance signals. In parallel, we encode textual prompts [19] of the form “Brand; Model; Type” (e.g., “BMW; X3; SUV”) using our proposed Vehicle Model Regulator and compare their t-SNE maps with those of the corresponding point cloud features (Figure 3a,b). The two distributions exhibit similar clustering patterns, which reveals a strong alignment between geometric features and text semantics, while also supporting our use of textual information as a high-level prior in the diffusion model.

To investigate whether the proposed Vehicle Model Regulator effectively captures semantic vehicle information, we visualize both textual semantic embeddings and point cloud feature embeddings using t-SNE, as shown in Figure 3a,b, respectively. Different colors correspond to different vehicle categories/types.

As observed in the visualizations, semantically related vehicle categories exhibit similar clustering tendencies in both the textual embedding space and the point cloud feature space. Representative categories such as SUVs, sedans, and pickup vehicles form relatively consistent neighborhood distributions across the two feature domains. This observation suggests that the proposed semantic regulator helps align textual semantic priors with geometric point cloud representations, thereby providing semantically meaningful guidance during the reconstruction process.

Our key idea is to embed rich prior knowledge into a diffusion-based point cloud generator so that the model can compensate for the information loss of single-view inputs and adhere to fine-grained vehicle semantics. Concretely, we design a prior-guided multimodal diffusion framework [20] in which **(i)** geometric priors from the input image (camera parameters, vehicle mask, distance-transform map, and vision backbone features) are projected onto the evolving 3D point cloud as control signals at each denoising step, providing strong structural cues under occlusion and viewpoint bias; **(ii)** semantic priors are introduced via textual prompts of the form “Brand; Model; Type”, whose CLIP embeddings are fused into the point cloud features through a cross-attention mechanism, guiding the generation toward vehicle-specific semantics; and **(iii)** a set of regulators, i.e., pretrained neural networks that encode high-level semantic priors, are used during training to regularize the diffusion process. The Vehicle Type Regulator learns the distribution of different vehicle categories and encourages type-consistent outputs, while the Vehicle Model Regulator aligns point cloud features with text features, suppressing mode collapse and semantic drift in low-information regimes. Intuitively, these regulators act as semantic critics that gently push the diffusion trajectory toward globally coherent and brand/model-consistent shapes.

Based on the above design motivations, our contributions are summarized as follows:

1. We investigate the problem of single-view vehicle point cloud reconstruction under non-ideal real-world conditions and analyze the limitations of existing general-purpose reconstruction frameworks in handling incomplete and semantically ambiguous vehicle observations.
2. We propose a prior-guided reconstruction framework that introduces semantic vehicle information, including brand, model, and category attributes, to improve semantic consistency and geometric plausibility during the point cloud generation process.
3. Extensive experiments demonstrate that the proposed framework achieves superior reconstruction quality and robustness compared with existing reconstruction approaches under challenging vehicle observation conditions.

## 2. Related Works

As previously discussed, 3D reconstruction from single-view inputs has emerged as an active and important research area in recent years.

### 2.1. Single Image to Point Cloud

Due to their sparsity and weak geometric constraints, point clouds have attracted widespread attention. Early methods for reconstructing complete point clouds from a single view primarily adopted encoder–decoder architectures. For example, the pioneering work 3D-R2N2 [21] employs a standard 2D convolutional network to encode the input 2D image into a low-dimensional embedding, processes this embedding using a 3D-LSTM [22], and then decodes it into a point cloud using a 3D convolutional network. Furthermore, LSM [23] first extracts feature maps from images using a 2D network and then lifts these features into a 3D point cloud representation, which is subsequently refined through a 3D convolutional network. Pix2Vox [24] and its enhanced version Pix2Vox++ [25] adopt a straightforward encoder–decoder framework, where a 2D convolutional encoder is paired with a 3D convolutional decoder, complemented by a multi-scale fusion mechanism. More recently, LegoFormer [26] introduces a transformer-driven method: it encodes the input image into a feature vector and reconstructs a dense voxel grid by generating sequences of small blocks. The decoding process relies on a non-autoregressive transformer decoder utilizing learned query embeddings. Inspired by the above works, in 2025, RGB2Point [27] proposed a transformer-based method for point cloud reconstruction, achieving the best performance among encoder–decoder-based approaches. In this paper, we regard RGB2Point [27] as the SOTA baseline for comparison with our proposed method. With the advancement of generative models, the PC2 [28] model has brought a disruptive change to this task. PC2 proposes a diffusion-based single-image point cloud reconstruction framework, where 2D encoded image features are projected into 3D space and used as conditional guidance during the denoising process for point cloud reconstruction. The design philosophy of PC2 inspired our model design for the proposed task. We regard PC2 [28] as our baseline and use it as a reference and comparison point in subsequent experiments. BDM [29] proposes a diffusion model based on Bayesian fusion of multiple models, claiming that the fused results outperform PC2 [28] on public datasets. We consider BDM [29] as the SOTA among fusion-based models and include it in our subsequent experimental comparisons. Although these methods achieve promising results in general object reconstruction, they are primarily designed for generic image-to-3D generation tasks and often rely heavily on visible image observations. Consequently, their reconstruction quality may significantly degrade under non-ideal vehicle scenarios involving occlusion, truncation, and incomplete structural information.

### 2.2. From Single Images to Other Forms of 3D Data

Recently, neural-field-based methods and implicit representations have also been widely explored for single-view 3D object reconstruction [30,31]. These approaches can achieve high-quality novel-view synthesis and geometric reconstruction, but they often rely on computationally expensive volumetric rendering and may suffer from instability under sparse-view or incomplete observation conditions. Apart from single-view 3D reconstruction methods that output point clouds, recent years have also seen the emergence of approaches that reconstruct meshes or adopt implicit representations. Works such as Nerf-WCE [32] and PixelNeRF [33] have developed NeRF-based [13] single-view reconstruction methods, which condition on image features from reference views and perform rendering through NeRF [13]. However, NeRF-based methods have been shown to have limitations, as discussed in [28]. In contrast, mesh-based methods have recently emerged as high-performance alternatives [34,35,36], with One-2-3-45++ [37,38] and Spar3d [39] being representative examples. One-2-3-45++ [38] generates consistent multi-view images by fine-tuning a 2D diffusion model, and it efficiently reconstructs textured 3D meshes by leveraging a 3D diffusion model. Spar3d [32] proposes an efficient 3D reconstruction method based on a diffusion model, which generates sparse point clouds and combines image information to quickly generate accurate 3D meshes. We took these two methods (One-2-3-45++ [38] and Spar3d [39]) as representative works based on other data formats and conducted comparative experiments. Existing point cloud reconstruction frameworks mainly focus on geometric generation and feature learning but generally do not explicitly incorporate semantic vehicle prior information such as brand, model, and category attributes. As a result, these methods may struggle to maintain semantic consistency and structural plausibility in ambiguous single-view reconstruction scenarios.

### 2.3. Asset Reconstruction Based on Non-Ideal Single View in Autonomous Driving Scenarios

As far as we know, VQA-Diff [40] is the only work that explores the reconstruction of vehicle assets from novel viewpoints by reconstructing assets from non-ideal perspectives. The authors relied on prior knowledge from LLMs to recognize and regenerate vehicles from existing non-ideal viewpoints. However, their work merely used prior knowledge from LLMs [41] to generate 2D novel viewpoints, rather than 3D assets with geometric information. Additionally, their work is only a pipeline, not a full model or algorithm. We reproduced their pipeline and compared it with our experimental results. Based on the above related works, we propose a neural network model that is different from and more novel and effective than the existing approaches [42]. We innovatively leverage prior information to improve both the input features and supervision of the model. Under the condition of non-ideal single-view input, we achieve more accurate vehicle point cloud asset reconstruction. In the following sections, we provide a detailed introduction to our proposed method and experimental results.

### 2.4. Discussion and Methodological Comparison

To further clarify the distinctions between the proposed framework and existing reconstruction approaches, we summarize the methodological characteristics of representative studies in Table 1.

Existing reconstruction frameworks mainly focus on recovering plausible geometry from single-view inputs, while largely ignoring fine-grained semantic consistency such as vehicle brand, model, and type. Encoder–decoder-based methods such as RGB2Point [34] primarily rely on direct feature mapping and often struggle under severely incomplete viewpoints. Diffusion-based methods such as PC2 [35] and BDM [36] improve geometric generation quality through iterative denoising, but they still lack explicit semantic constraints.

Meanwhile, methods such as PixelNeRF [38], One-2-3-45++ [23], and Spar3D [18] mainly focus on novel-view synthesis or mesh reconstruction, rather than semantically consistent vehicle asset generation under non-ideal viewpoints. VQA-Diff [43] introduces LLM-based prior reasoning; however, it relies heavily on the correctness of external semantic inference and does not directly constrain 3D geometric generation.

In contrast, our method jointly incorporates geometric priors, text-guided semantic priors, and frozen semantic regulators into a unified diffusion framework. This design enables the model to maintain both geometric plausibility and semantic consistency under severely under-constrained single-view conditions, thereby improving robustness against occlusion, truncation, and viewpoint ambiguity.

In summary, despite recent advances in image-to-3D and point cloud reconstruction, existing methods still suffer from two important limitations for real-world vehicle reconstruction tasks: (1) insufficient utilization of semantic vehicle prior information, and (2) limited robustness under non-ideal single-view observations with incomplete structural visibility. These limitations motivate the proposed prior-guided semantic reconstruction framework.

## 3. Methodology

### 3.1. Overview

Although point clouds provide a flexible and lightweight geometric representation for vehicle reconstruction, they are not directly suitable for high-fidelity rendering in simulation environments. In practical applications, the reconstructed point clouds can be further integrated with downstream surface reconstruction or mesh generation methods, such as Poisson surface reconstruction, neural surface fitting, or Gaussian-splatting-based rendering frameworks, to generate renderable vehicle assets. Compared with directly reconstructing meshes under incomplete observations, point clouds impose weaker topological constraints and, therefore, provide greater robustness under occlusion and non-ideal viewpoints.

Vehicle point cloud reconstruction from casually captured single-view images is particularly challenging due to severe occlusion, incomplete structural visibility, arbitrary viewpoints, and semantic ambiguity between visually similar vehicle categories. Therefore, an effective reconstruction framework should simultaneously model geometric consistency and semantic structural priors.

**Figure 4:**
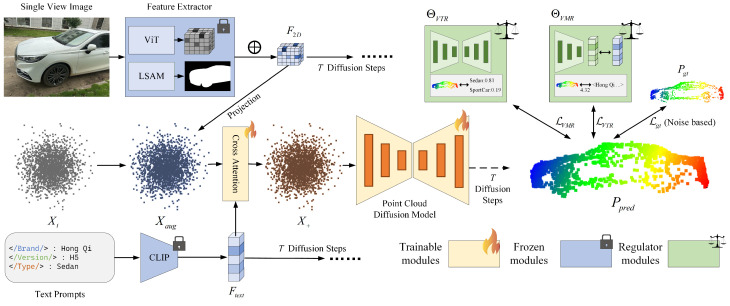
The overall pipeline of the proposed model. A single-view image and the corresponding textual prompts serve as inputs, while the reconstructed point cloud is generated as the output. The proposed model is a multimodal conditional diffusion model. At each denoising step, every point in the point cloud is enhanced by its corresponding projected 2D features and text features. During the training process, in addition to the ground-truth supervision, the output is also regulated by the regulators.

We propose a prior-guided diffusion model $S_{\theta }\in R^{3\times N}\to R^{3\times N}$ for vehicle point cloud reconstruction from non-ideal monocular image inputs. The single-view image $I\in R^{H\times W\times 3}$ and the corresponding textual prompts T serve as inputs, while the reconstructed point cloud $P_{pred}\in R^{N\times 3}$ is generated as the output. As shown in Figure 4, the inputs are used as conditioning signals for the diffusion model, serving as enhanced features of the point cloud at each denoising step. During the training phase, apart from the predefined noise $\epsilon \in R^{3\times N}$, we also supervise the reconstructed point cloud $P_{pred}$ through pretrained regulators.

### 3.2. Architecture

#### 3.2.1. Prior-Guided Multimodal Conditional Diffusion Model

The diffusion model is a general generative model based on stochastic differential equations and nonequilibrium thermodynamics principles. The proposed multimodal diffusion model $S_{\theta }$ can be introduced from two aspects: the training phase and the sampling phase. In the training phase, we denote the ground-truth point cloud $P_{gt}\in R^{3\times N}$ as $X_{0}$. As shown in Equation (1), $X_{0}$ undergoes *t* iterations of periodic noise addition, resulting in $X_{t}$.

(1) $$\rho \left(X_{t}|X_{0}\right)\;=\;\sqrt{{\bar{\alpha }}_{t}}\;X_{0}\;+\;\sqrt{{1-\bar{\alpha }}_{t}\;}\;\epsilon$$

where ρ(•) represents the data distribution, and ${\bar{\alpha }}_{t}\;=\;{⨿}_{i=1}^{t}\left(1-\beta_{i}\right)$ and $\beta_{i}\in \left[0,1\right]$ are predefined variance parameters of Gaussian distribution; ϵN(0,I) is a Gaussian noise. Due to the noise distribution at each step of the noise addition process being known, the transition between two adjacent noise steps can be expressed as shown in Equation (2).

The role of the prior-guided multimodal conditional diffusion model $S_{\theta }$ is to predict the reverse process $\rho (X_{t-1}|X_{t})$. During the training phase, the diffusion model takes the noisy input $X_{t}$, along with the prior information *C* (the detailed process and implementation are elaborated in Section 3.2.2) related to $X_{t}$. We denote this process as $S_{\theta }\left(X_{t-1}|X_{t},\;C\right)\to \;\;\rho \left(X_{t-1}|X_{t}\right)$.

Through *t* steps of the reverse process, i.e., the denoising process, we obtain the predicted noise $\epsilon_{pred}\in R^{3\times N}$. During the training phase, $\epsilon_{pred}$ is supervised using the ground-truth ϵ and regulators. The details of the regulators and supervision process are provided in Section 3.3. Once the predicted noise $\epsilon_{pred}$ is obtained, the reconstructed or predicted point cloud $P_{pred}$ can be derived using the formula shown in Equation (3).

(2) $$\rho \left(X_{t}|X_{t-1}\right)\;=\;N\left(X_{t};\;\sqrt{{1-\beta }_{t}\;}X_{t-1},\beta_{t}I\right)$$

(3) $$P_{pred}\;=\;\frac{X_{t}\;-\;\sqrt{{1-\bar{\alpha }}_{t}\;}\epsilon_{pred}}{\sqrt{{\bar{\alpha }}_{t}}}$$

During the sampling phase, the single-view image and text prompt remain as the conditioning signal *C*, while $X_{t}$ is a randomly sampled Gaussian noise, $X_{t}\;N\left(0,I_{3\times N}\right)$. After *t* steps of denoising, we obtain the reconstructed point cloud $P_{pred}$, conditioned on *C*. In the experimental process, two sampling strategies (DDPM [44] and DDIM [43]) are tested.

#### 3.2.2. Prior Information Control

This section primarily provides a detailed pipeline describing the role of *C* in the process of $S_{\theta }\left(X_{t-1}|X_{t},\;C\right)$ mentioned in Section 3.2.1. As mentioned in the Section 1, we incorporate prior information to enhance the noisy point cloud input during the denoising process. Our enhancement approach is as follows: we use the single-view input image and corresponding camera parameters (intrinsic and extrinsic) to provide geometric prior guidance for the noisy point cloud. To address challenges such as complex backgrounds, we also treat the vehicle mask in the image, along with the quantization of the distance from each point in the image to the nearest pixel of the mask, as an additional channel in the image input. We aim to leverage the prior mask information to help the neural network focus more on the specified vehicle target. In addition to the image features, based on our findings, we extract the text prompt as feature embeddings using CLIP [19]. These embeddings are then fused with the noise-enhanced point cloud through a cross-attention mechanism, providing prior information guidance for the subsequent point cloud generation.

Formally, for the monocular image input $I\in R^{H\times W\times 3}$, we obtain the mask of the specified vehicle, denoted as $M\in R^{H\times W}$, using LSAM [45]. For the mask *M*, we calculate the Euclidean distance $M\in R^{H\times W}$ of each pixel to the nearest valid point $V=\left(x_{q},y_{q}\right)|M\left(x_{q},y_{q}\right)=1$, as shown in Equation (4):

(4) $$D\left(p\right)={Norm(min}_{q\in V}{\left\|p-q\right\|}_{2})$$

where *p* represents a pixel in the mask, and D(p) is a normalized and clipped Euclidean distance quantization, which is also incorporated as part of the prior information to guide the model $S_{\theta }$. For the image *I* itself, we use ViT to extract the features of *I*, resulting in $F_{rgb}\in R^{H\times W\times F1}$. We concatenate all of the 2D prior information together, and we denote it as $F_{2D}=\mathrm{Concat}\left(I,\;M,\;D,\;F_{\mathrm{rgb}}\right)\in R^{H\times W\times (3+1+1+F_{1})}$ (*F*1 is set to 384 in this paper).

For the output point cloud $X_{t}$ at each step *t* in the denoise phase, we project the 2D prior information $F_{2D}$ into the 3D space using a rasterizing projection operation ${F_{3D}\;=\;P}_{r}{(X}_{t},F_{2D}$).

The $F_{3D}\in R^{(5+F1)\times N}$ feature is concatenated with the point cloud $X_{t}$ at the current *t*-th step; we denote it as $X_{aug}=Concat\left(F_{3D},X_{t}\right)\in R^{(5+F1+3)\times N}$. At this point, the noisy point cloud $X_{aug}$ is enhanced with the information from the single-view image input, and then we further enhance $X_{aug}$ using the text-based prior information. We use the pretrained CLIP model to extract features from the text information corresponding to the image, denoted as $F_{text}\in R^{F2}$ (F2 is set to 512 in this paper). We then fuse $X_{aug}$ and $F_{text}$ through a fully connected layer and the cross-attention structure, denoted as $\;X_{+}=CrossAtten\left(X_{aug},\;F_{text}\right)\in R^{(5+F1+3)\times N}$. Thus, process $S_{\theta }\left(X_{t-1}|X_{t},\;C\right)$ can also be represented as process $S_{\theta }\left(X_{t-1}|X_{+}\right)$.

**Figure 5:**
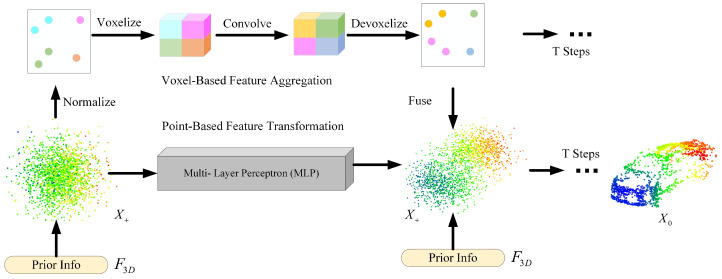
The structure of our diffusion model. It consists of two parallel branches, enabling the fusion of global and local features. The voxel branch consists of a series of 3D convolutions, while the point cloud branch is composed of MLPs.

As shown in Figure 5, we process the point cloud $X_{t}$ using two parallel branches: the point-based branch, which independently processes individual points using Multilayer Perceptrons (MLPs) [46]; and the voxel-based branch, which aggregates local features through 3D convolutions on a low-resolution voxel grid. The voxel features are then mapped back to the point cloud using trilinear interpolation, ensuring spatial alignment. By combining these two branches, the proposed diffusion model effectively captures both global and local structures while maintaining computational efficiency.

The overall procedure of the prior-guided conditional denoising at each diffusion step is summarized in Table 2.

### 3.3. Regulators and Supervision

This section primarily introduces the design of the regulators and the supervision mechanism in our proposed model.

#### 3.3.1. Regulators

We propose a novel strategy by pretraining neural networks as regulators to learn the characteristics and distribution of existing data. This enables the diffusion model to be guided by high-dimensional prior features effectively. Under non-ideal vehicle observation conditions, visually similar local regions may correspond to structurally different vehicle categories, making purely appearance-driven reconstruction highly ambiguous. To alleviate this issue, we designed two types of regulators: the Vehicle Type Regulator $\Theta_{VTR}$ explores the distribution characteristics of point clouds corresponding to different vehicle types (e.g., SUV, Sedan, etc.) and supervises the point clouds generated by the diffusion model $S_{\theta }$; the Vehicle Model Regulator $\Theta_{VMR}$ identifies the relationship between text-prompt features and their corresponding point clouds, supervising the diffusion model to generate point clouds that are closer to the associated textual prompt.

**Figure 6:**
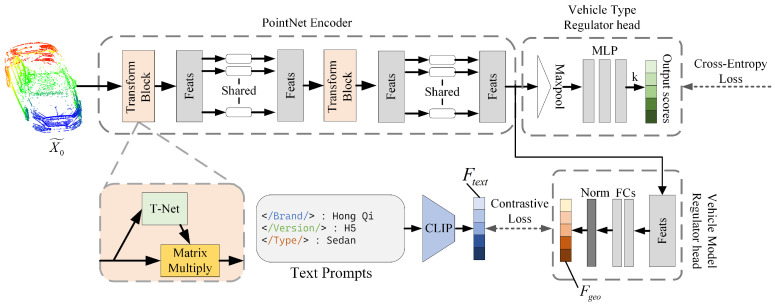
The structures of the Vehicle Type Regulator and Vehicle Model Regulator. Both regulators take the augmented ground-truth point cloud as input and encode it using a combination of transformer blocks and MLPs. The Vehicle Type Regulator is designed to learn the data distribution characteristics across different vehicle types, while the Vehicle Model Regulator captures the association between the point cloud data and text prompts.

As shown in Figure 6, all of the regulators take the ground-truth point cloud ${\tilde{P}}_{gt}\in R^{3\times N}$ as input. The point clouds $P_{gt}\in R^{3\times N}$ undergo random augmentations such as scaling, rotation, translation, and index shuffling. We select the PointNet [17] Encoder as the encoder for the regulators. For the $\Theta_{VTR}$, we appended a max-pooling layer followed by three MLP layers after the PointNet Encoder to output the classification result for the input ${\tilde{P}}_{gt}$. We labeled the $P_{gt}$ into “Sedan,” “SUV,” “Pickup,” “MPV,” “SportsCar,” “Van,” “Truck,” “Bus,” and “EV”; these labels are marked as $C_{gt}\in R^{1\times N_{cls}}$. During the training of the $\Theta_{VTR}$, we used the cross-entropy loss, as shown in Equation (5):

(5) $$L_{CE}\left(C_{gt},\Theta_{VTR}\left({\tilde{P}}_{gt}\right)\right)\;=\;-\sum_{i=1}^{N_{cls}}C_{gt,i}{log(\Theta_{VTR}\left({\tilde{P}}_{gt}\right))}_{i}$$

where $N_{cls}$ represents the number of vehicle type categories. Unlike the $\Theta_{VTR}$, for the $\Theta_{VMR}$, we use two fully connected layers to align the features output by the encoder with the text features $F_{text}\in R^{F2}$. Each fully connected layer is followed by a LayerNorm layer, and the first fully connected layer includes a sigmoid activation function. We train the $\Theta_{VMR}$ by a contrastive loss, and this process is shown in Equation (6):

(6) $$\begin{array}{cc} L_{\mathrm{Conj}}\left(F_{\mathrm{text}},\Theta_{\mathrm{VMR}}\left({\tilde{P}}_{gt}\right),Y_{L}\right)= & Y_{L}\cdot {∥F_{\mathrm{text}}-\Theta_{\mathrm{VMR}}\left({\tilde{P}}_{gt}\right)∥}_{2}^{2}\\ & +\left(1-Y_{L}\right)\cdot \max\left(0,\;m-{∥F_{\mathrm{text}}-\Theta_{\mathrm{VMR}}\left({\tilde{P}}_{gt}\right)∥}_{2}^{2}\right) \end{array}$$

where $Y_{L}$ is the label indicating whether the feature embedding $F_{text}$ and the $\Theta_{VMR}\left({\tilde{P}}_{gt}\right)$ are matched. We generate positive and negative samples by shuffling a batch of training data. The hyperparameter *m* represents the margin in the $L_{Conj}$ (we set *m* = 1 in this paper). Once all of the regulators are trained, they are frozen and no longer updated. During the training phase of the $S_{\theta }$, these regulators take the $P_{pred}\in R^{3\times N}$ as input. Their inference results then serve as part of the supervisory loss for training the $S_{\theta }$.

From a regularization perspective, the frozen regulators play a similar role to auxiliary tasks in multi-task learning and teacher models in knowledge distillation. In both settings, additional structured signals act as inductive biases that constrain the hypothesis space and improve generalization, either by enforcing shared representations across related tasks or by encouraging the student to match a fixed teacher’s predictions or embeddings [47,48,49]. Analogously, our pretrained Vehicle Type and Vehicle Model Regulators impose semantic constraints on the generated point clouds, guiding the diffusion model toward solutions that are consistent with high-level type distributions and text–geometry alignments learned from the full dataset, rather than overfitting to the limited supervision of single-view inputs.

#### 3.3.2. Supervision

According to the description in Section 3.2, our designed diffusion model $S_{\theta }$ directly outputs the predicted noise $\epsilon_{pred}=S_{\theta }\left(X_{0}|X_{t},\;C\right)\in R^{3\times N}$, from which the reconstructed point cloud $P_{pred}\in R^{3\times N}$ can be obtained using Equation (3). Therefore, our supervision is divided into two main parts: One part involves directly supervising the $\epsilon_{pred}$ using the ground-truth noise $\epsilon_{gt}=\rho \left(X_{0}|X_{t}\right)\in R^{3\times N}$. This process is implemented with L2 loss, as formulated in Equation (7).

(7) $$L_{gt}=\;\|{\epsilon_{gt}-\epsilon_{pred}\|}_{2}^{2}$$

On the other hand, for the reconstructed $P_{pred}$, we input it into the regulators for supervision. For the Vehicle Type Regulator $\Theta_{VTR}$, we apply the cross-entropy loss for supervision, similar to Equation (5). This process is formulated in Equation (8):

(8) $$L_{\mathrm{VTR}}\left(C_{gt},\;\Theta_{\mathrm{VTR}}^{*}\left(P_{pred}\right)\right)=-\sum_{i=1}^{N_{\mathrm{cls}}}C_{gt,i}\cdot \log\left({\left[\Theta_{\mathrm{VTR}}^{*}\left(P_{pred}\right)\right]}_{i}\right)$$

where the ∗ in $\Theta_{VTR}^{*}$ indicates that the neural network weights are frozen and no longer updated. It is worth noting that $L_{CE}$ is not used for training the regulator $\Theta_{VTR}$. Instead, it guides the training of the diffusion model $S_{\theta }$ by leveraging the output of $\Theta_{VTR}^{*}$ on $P_{pred}$. For example, if the type label of the ground truth is “SUV,” we expect the regulator $\Theta_{VTR}^{*}$ to classify $P_{pred}$ as closer to “SUV.” This is the fundamental difference between Equations (8) and (5).

For the Vehicle Model Regulator $\Theta_{VMR}$, we use the matching score between the text feature $F_{text}\in R^{F2}$ and the point cloud feature $\Theta_{VMR}^{*}\left(P_{pred}\right)\in R^{F2}$, which is obtained by inputting $P_{pred}$ into the $\Theta_{VMR}^{*}$, as the supervision signal, as shown in Equation (9):

(9) $$L_{\mathrm{VMR}}\left(F_{\mathrm{text}},\;\Theta_{\mathrm{VMR}}^{*}\left(P_{\mathrm{pred}}\right)\right)=\frac{1}{F_{2}}\sum_{i=1}^{F_{2}}{∥F_{\mathrm{text},i}-{\left[\Theta_{\mathrm{VMR}}^{*}\left(P_{\mathrm{pred}}\right)\right]}_{i}∥}_{2}^{2}$$

As shown in Equation (9), we use the mean trace of $F_{text}$ and feature $\Theta_{VMR}^{*}\left(P_{pred}\right)$ to represent their matching score, which guides the $S_{\theta }$ to generate a point cloud $P_{pred}$ that better aligns with the textual semantics. For example, if the text prompt is (BMW, X3, SUV), the $\Theta_{VMR}^{*}$, as a frozen regulator, extracts features from $P_{pred}$. By supervising the L2 loss between this feature $\Theta_{VMR}^{*}\left(P_{pred}\right)$ and the text feature $F_{text}$, we guide $P_{pred}$ to better conform to the textual semantics.

(10) $$L_{\mathrm{total}}=\lambda_{1}L_{\mathrm{gt}}+\lambda_{2}L_{\mathrm{VTR}}\left(C_{gt},\;\Theta_{\mathrm{VTR}}^{*}\left(P_{pred}\right)\right)+\lambda_{3}L_{\mathrm{VMR}}\left(F_{\mathrm{text}},\;\Theta_{\mathrm{VMR}}^{*}\left(P_{pred}\right)\right)$$

In summary, during the training of the $S_{\theta }$, the total loss $L_{total}$ can be expressed in the form of Equation (10), where $\lambda_{1}$, $\lambda_{2}$, and $\lambda_{3}$ are hyperparameters that are responsible for maintaining the balance of the supervision strength.

## 4. Dataset Construction

This section mainly discusses the establishment and analysis of our dataset. To the best of our knowledge, 3DRealCar [50] is the first and currently the only large-scale 3D real car dataset, which contains 2500 car instances and their point clouds with actual sizes in real-world scenes. Based on 3DRealCar, we curate a higher-quality dataset, 3DRealCar++, tailored for fine-grained vehicle point cloud reconstruction.

The 3DRealCar dataset was collected using image-based 3D reconstruction methods such as COLMAP [51] and SAM [52]. However, this pipeline suffers from two key limitations: (1) the lack of accurate camera parameters and depth data introduces errors in pose estimation and point cloud generation; (2) semantic segmentation with SAM requires extensive manual validation to ensure the correctness of masks, which is labor-intensive and error-prone. We observed a large number of inaccurate masks and reconstruction artifacts in the dataset, as COLMAP fails to detect or correct erroneous inputs, instead propagating these errors throughout the reconstruction process.

**Figure 7:**
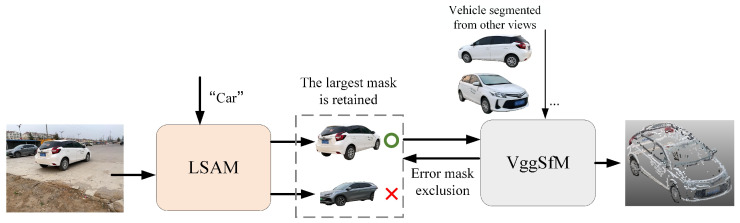
Pipeline of 3DRealCar++ construction. LSAM [45] is used to extract and filter vehicle masks, which are then fed into VggSfM [53] for point cloud reconstruction. The resulting high-quality point clouds serve as the ground truth for all images of the same instance.

Therefore, in 3DRealCar++, we optimize this process. The overall data curation and reconstruction pipeline is illustrated in Figure 7. For each image used for SfM [51], we employ LSAM [45] with the prompt “car” to perform segmentation, ensuring that all extracted masks correspond to vehicles.

We feed the images and the corresponding masks into VggSfM [53] for vehicle point cloud reconstruction. Additionally, based on the reprojection filtering functionality of VggSfM [53], the masks segmented by LSAM [45] undergo further filtering. Masks and their corresponding images with too few reprojected points are considered invalid and are discarded.

We then perform a final round of filtering for the reconstructed point clouds and corresponding images. Point clouds with fewer than 2048 points are considered invalid and discarded. For each valid point cloud, we manually check for errors and remove any erroneous reconstructions. Additionally, we review the quality of the corresponding images (e.g., lighting, motion blur) and discard those with poor quality. The remaining high-quality images, along with their camera parameters, are retained as monocular inputs, with each valid point cloud corresponding to multiple high-quality images.

**Figure 8:**
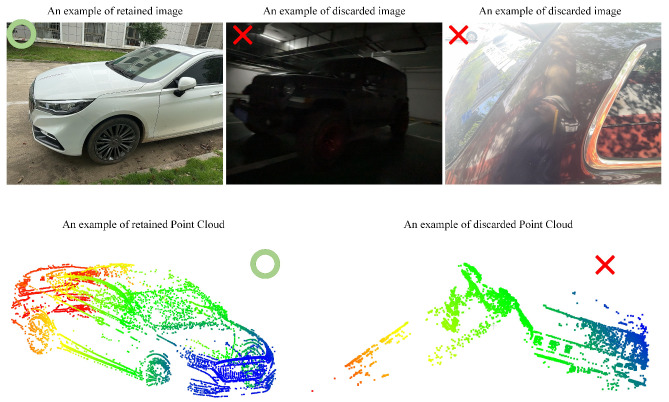
Examples of our dataset filtering process. High-quality data is retained, while low-quality or erroneous data is discarded.

**Figure 9:**
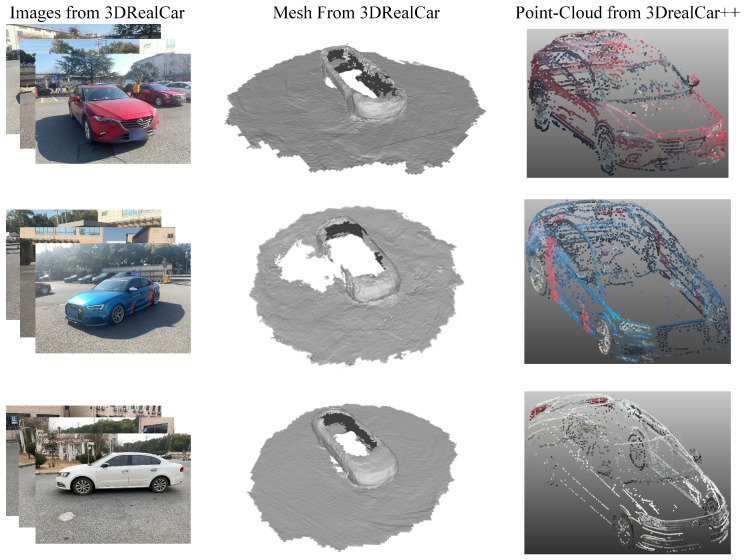
Qualitative comparison between the original 3DRealCar dataset and our optimized 3DRealCar++.

Figure 8 illustrates our dataset filtering process. A comparison between the original 3DRealCar and our optimized 3DRealCar++ is shown in Figure 9. The comparison reveals that the original dataset’s reconstructed point clouds suffer from background noise and poor uniformity, with ground points surrounding the vehicle. In contrast, while our point clouds remain sparse, their uniformity and accuracy are significantly improved.

As introduced in Section 1, we assign textual descriptions to each instance using human annotation and LLM assistance (ChatGPT-4o API). For each instance, the most distinctive image is selected, and the LLM is queried for the vehicle’s brand, model, and type in the format “Brand; Model; Type” (e.g., “BMW; X3; SUV”). The generated descriptions are then manually reviewed for accuracy.

Table 3 presents the distribution and statistical results of our 3DRealCar++ dataset. After filtering, 2017 vehicle instances from the original 3DRealCar dataset were retained, each associated with multiple surrounding-view images and annotated for brand, model, and type.

To address the data imbalance (e.g., fewer buses compared to sedans), we applied augmentation techniques (flipping, rotation, scaling) for underrepresented types and randomly removed data from overrepresented types. The 3DRealCar++ dataset was split into 60% training (14,335 images) and 40% testing (9554 images), ensuring no overlap between the sets.

## 5. Experiments

### 5.1. Implemention Details

During training and evaluation, each image was resized to 256 × 256, and each point cloud was downsampled to 2048 points. Point clouds underwent random rotation, translation, scaling, and shuffling during training to improve generalization, while no augmentation or shuffling was applied during evaluation for consistency.

Regarding the training of the Vehicle Type Regulator $\Theta_{\mathrm{VTR}}$, we employed the Adam optimizer with an initial learning rate of $5\times 10^{-4}$. The optimizer was configured with $\beta_{1}=0.9$ and $\beta_{2}=0.999$, an epsilon value of $1\times 10^{-8}$, and a weight decay of $1\times 10^{-4}$ to prevent overfitting. The model was trained for 500 epochs using the designated training split of 3DRealCar++.

For the Vehicle Model Regulator $\Theta_{VMR}$, we used the same optimizer as $\Theta_{VTR}$, but we set the initial learning rate to 1 × 10^−3^ to achieve better convergence and stability based on empirical observations. Additionally, a learning rate scheduler was employed, reducing the learning rate by a factor of 0.7 every 20 epochs, aiming to stabilize training and promote convergence.

For the proposed diffusion model, we adopted a standard generative model training strategy. The model was trained for 100,000 iterations, with a batch size of 16 per training step. We used the AdamW optimizer with $\beta_{1}=0.9$ and $\beta_{2}=0.999$ [28]. Throughout the training process, we set the initial learning rate to $2\times 10^{-4}$ and applied linear decay to 0 over the course of the training steps.

For the hyperparameters in the loss function mentioned in Section 3.3.1, we set $\lambda_{1}=1$, $\lambda_{2}=0.1$, and $\lambda_{3}=0.05$. The hyperparameter settings for the loss function mainly depend on the magnitude of each loss term. We aimed to keep their magnitudes and convergence speeds as consistent as possible.

For our diffusion noise schedule, we used a linear schedule with a warm-up phase, where β linearly increases from $1\times 10^{-5}$ to $8\times 10^{-3}$.

For the sampling phase, we propose two sampling modes based on DDPM [44] and DDIM [43]. During the evaluation phase, similar to previous state-of-the-art works like PC2 [28], BDM [29], and PVD [54], we chose the F-score and Chamfer Distance [29] as the quantitative evaluation metrics. Our evaluation code is consistent with that of BDM [29], and more details can be found in their open-source code.

All experiments were conducted on a single NVIDIA RTX 3090 GPU. The code was primarily implemented in Python 3.9, with the core model built using PyTorch 2.0, and rasterization projections handled by PyTorch3D 0.7.4.

### 5.2. Semantic Annotation Workflow Discussion

The proposed semantic priors are annotated at the vehicle-instance level rather than the image level. Since multiple images correspond to the same reconstructed vehicle instance, only one semantic label of the form “Brand; Model; Type” is required for an entire image group. This significantly reduces the annotation burden compared with conventional image-wise labeling pipelines.The average human verification time for each vehicle instance is approximately 15 s.

To further improve efficiency, we employed an LLM-assisted annotation strategy using the ChatGPT-4o API to automatically generate candidate semantic descriptions. Human annotators mainly performed lightweight verification and correction instead of manually labeling all semantic information from scratch.

Therefore, the overall human workload remained manageable, while preserving semantic consistency across the dataset. Moreover, in practical deployment scenarios, semantic priors can be automatically estimated using existing vehicle recognition systems, OCR-based identification methods, or vision–language models, reducing the dependence on manual annotation during inference.

### 5.3. Comparison with Existing Methods

For point cloud-based reconstruction methods, we selected RGB2Point [27], PC2 [29], and BDM [32] as strong baselines, and we further included two recent diffusion-based 3D reconstruction approaches—SDFit [55] and ICDDPM [56]—for a comprehensive comparison with our method. We reimplemented all methods and followed the training settings reported in their original papers as closely as possible. To ensure fairness, however, every model was trained for the same number of iterations (100,000) on 3DRealCar++. The quantitative results under this unified protocol are summarized in Table 4.

Following several recent diffusion-based point cloud reconstruction frameworks [29], this work primarily adopts the Chamfer Distance (CD) and F-score for quantitative evaluation. Compared with the Earth Mover’s Distance (EMD), CD is more suitable for measuring the geometric coverage and structural consistency of generated point clouds under stochastic sampling conditions. Since the proposed task focuses on semantically plausible vehicle reconstruction rather than strict point-wise correspondence, CD-based evaluation provides a more computationally practical and structurally representative assessment for large-scale diffusion-based reconstruction experiments.

As shown in Table 4, our DDIM-based variant (Ours) already surpasses all baselines in terms of both F1-score and Chamfer Distance, improving the F1-score from 0.6163 (BDM) to 0.6990 and reducing the CD from 79.2421 to 70.0585. When switching to the DDPM sampling strategy (Ours-DDPM), performance is further boosted across all metrics: precision increases from 0.7020 to 0.7399, recall from 0.7278 to 0.7395, and F1-score from 0.6990 to 0.7226, while the Chamfer Distance decreases from 70.0585 to 62.1525. While DDPM sampling delivers the best reconstruction quality, it also entails higher computational cost; therefore, all main comparisons with prior SOTA methods and most ablation studies are reported using the DDIM-based variant, with DDPM results provided to reveal the upper-bound potential of our model under more expensive sampling settings.

Furthermore, as shown in Table 4, by adopting the DDPM sampling strategy, our method (Ours-DDPM) achieves further improvements across all metrics compared to the DDIM-based version (Ours), with precision increasing from 0.7020 to 0.7399, recall from 0.7278 to 0.7395, and F-score from 0.6990 to 0.7226, while the Chamfer Distance decreases from 70.0585 to 62.1525. Although DDPM-based sampling yields superior performance, it also incurs significantly higher computational costs. Therefore, all major comparisons with prior SOTA methods and ablation studies are conducted based on the DDIM-based results. The DDPM-based results are provided to demonstrate the further potential of our model under more powerful computational settings.

**Figure 10:**
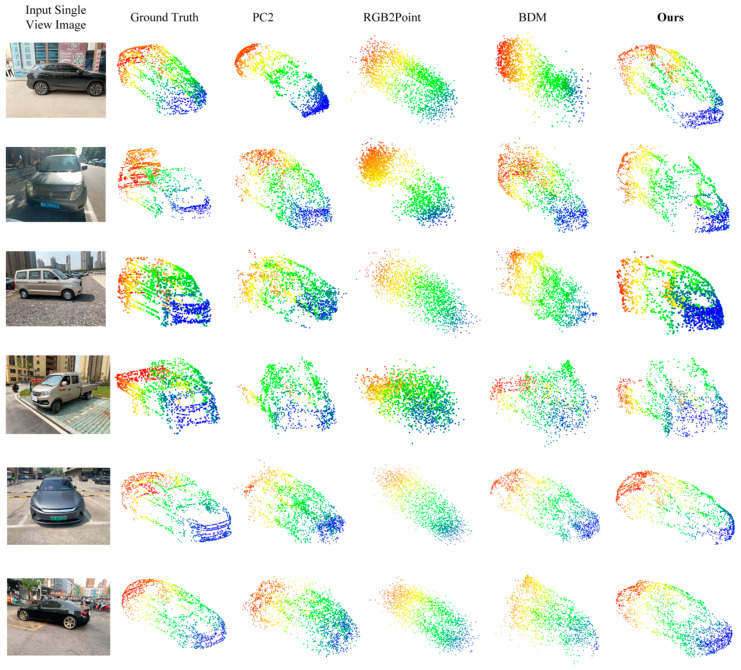
Comparison between our model and state-of-the-art point cloud-based reconstruction methods.

In Table 5, we provide detailed quantitative results for each vehicle category. Although data balancing techniques were applied during training, we can observe that model performance across categories remains closely correlated with the original sample distribution—categories with fewer real samples, such as Van and Sport Car, generally exhibit lower F-scores. Nevertheless, the overall performance, with an average F-score of 0.6990 achieved by our model, highlights its superiority over existing state-of-the-art approaches. Additionally, we provide several representative cases in Figure 10 for qualitative analysis.

From the results shown in Figure 10, our method reconstructs point clouds that most accurately approximate the ground-truth geometry, further validating the objectivity of our quantitative results. Combining both quantitative and qualitative analyses, we present the following discussions:

RGB2Point adopts a transformer-based encoder–decoder architecture; however, under non-ideal conditions, its performance falls short compared to diffusion-based models such as PC2 and BDM, which leverage iterative denoising. Nevertheless, the performance of PC2 remains significantly constrained due to the lack of prior information guidance. Although BDM incorporates a joint reasoning paradigm between two models, it also struggles to achieve robust performance when sufficient input information is unavailable.

**Figure 11:**
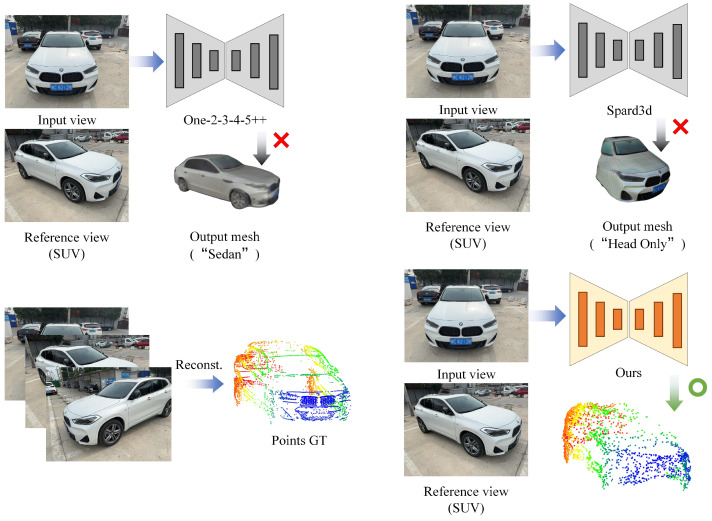
Case studies comparing our point cloud-based single-view reconstruction method with other end-to-end single-view reconstruction methods under non-ideal viewpoints. It can be observed that, due to limited visibility or missing information, general-purpose methods may not be effective under non-ideal conditions.

In addition to the quantitative comparisons against point cloud-based reconstruction methods, we also compare our approach with other end-to-end monocular 3D reconstruction methods. As shown in Figure 11, we provide several case studies aimed at theoretically validating the robustness of our prior-based approach under non-ideal conditions, rather than relying solely on qualitative results.

Specifically, in Figure 11, we select two of the most prominent end-to-end mesh reconstruction methods—One-2-3-45++ [38] and Spar3d [39]—to benchmark against our approach. While these methods are highly effective for general reconstruction tasks, they can still produce notable errors when the input image contains limited visual information. For instance, One-2-3-45++ reconstructs an SUV as a sedan when only the front of the vehicle is visible, while Spar3D merely reconstructs a partial frontal section of the car.

From a narrow, image-consistency perspective, these results are technically correct, as they form a closed loop with the available image data. However, from the broader perspective of controllable asset generation for autonomous driving applications, such errors are critical. The root cause of these mistakes lies in the inherent limitations of single-view input images. In contrast, our prior-guided point cloud reconstruction method, informed by prior knowledge, successfully reconstructs the accurate 3D geometry of vehicles, even under information-sparse conditions.

**Figure 12:**
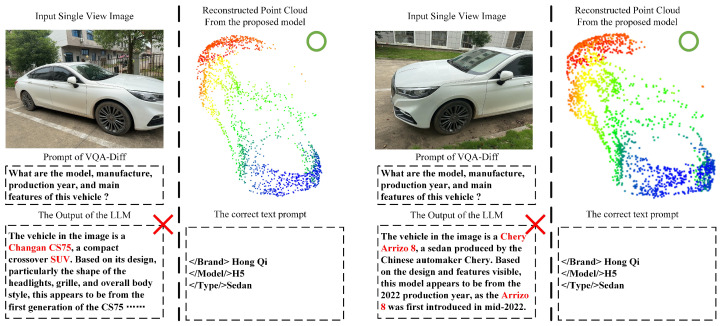
A comparison of several case studies with the LLM-based VQA-Diff method. Due to the hallucination issues of LLMs, such methods may be unreliable under non-ideal conditions.

Furthermore, VQA-Diff [40] introduces an LLM-based [41] approach that leverages the extensive knowledge and reasoning capabilities of LLMs to perform 3D reconstruction from single-view image inputs. We reproduced the LLM-related component of their work using ChatGPT-4o, and the results are shown in Figure 12.

Due to the hallucination issues inherent in current LLMs, their responses under suboptimal input conditions are not always accurate. As shown in Figure 12, both input images depict a “Hong Qi” brand vehicle of the “H5” model. However, the LLM fails to provide the correct identification, leading to inaccurate downstream reconstruction by VQA-Diff [40].

Therefore, we argue that guiding 3D reconstruction under constrained conditions using prior information is a more reliable and robust approach.

### 5.4. Ablation Studies

This section presents ablation studies to verify the importance and effectiveness of the key components in our method. Each experiment isolates a specific factor to clarify its contribution to the final performance.

We ablate the two regulators, $\Theta_{VTR}$ and $\Theta_{VMR}$, introduced in Section 3.3.1. The results in Table 6 show that both modules exhibit improved performance, with $\Theta_{VTR}$ providing a more pronounced gain. This is likely because features associated with different vehicle types are more separable in the feature space, offering stronger guidance for generating point clouds of the desired category.

We evaluate the feature fusion strategy described in Section 3.2.2. In particular, we study the effect of incorporating text features and the choice of cross-attention for fusion. As summarized in Table 7, introducing text features already leads to a clear performance boost (first vs. third column), and replacing simple concatenation with attention-based fusion yields further improvements (second vs. third column), confirming that our fusion design is both necessary and effective.

In addition to the fusion mechanism, we also examined the impact of different text encoders on our framework. We compared the original CLIP text encoder with two widely used language models—BERT [57] and RoBERTa [58]—as well as a baseline without any text priors. As shown in Table 8, using text priors consistently improves performance over the no-text setting, and CLIP provides the strongest gains among all choices, likely due to its stronger alignment between visual and textual semantics.

We further investigated the effects of different diffusion noise schedules while keeping the sampling strategy fixed. In particular, we compared a linear schedule with a warm-up (our default choice) against cosine and quadratic schedules. As reported in Table 9, all three schedules yield reasonable results, but the linear schedule consistently achieves the best F1-score and the lowest Chamfer Distance, justifying our design choice in Section 4.

To better understand the role of the regulators as semantic regularizers, we compared different training schemes: (i) removing both regulators, (ii) jointly training regulators and the diffusion model end-to-end, and (iii) using our two-stage strategy where the regulators are pretrained and then frozen. As summarized in Table 10, joint training improves over the no-regulator setting but still underperforms our frozen-regulator scheme, which achieves the best F1-score and the lowest Chamfer Distance. This supports our interpretation that frozen regulators act as stable, data-dependent regularizers rather than merely adding model capacity.

We also compared different sampling strategies for the diffusion model, as reported in Table 11. The first row corresponds to DDPM sampling with 100 steps from random Gaussian noise, the second row to DDIM sampling with 100 steps, and the third row to DDPM with 1000 steps. Consistent with expectations, DDPM with 1000 steps achieves the best reconstruction quality, but at a substantial cost in inference time. Therefore, we adopted DDIM with 100 steps as our default setting, which provides a favorable trade-off between accuracy and efficiency.

Finally, we designed a reverse experiment to further validate the effectiveness and necessity of the regulators. We fed four types of point clouds—Gaussian noise, outputs from PC2, our reconstructed results, and ground-truth point clouds—into the regulators and compared their evaluations. As shown in Table 12, the Vehicle Type Regulator (VTR) was assessed using instance accuracy, while the Vehicle Model Regulator (VMR) was evaluated using the matching score, defined as the Euclidean distance in Equation (9). Gaussian noise yielded the worst scores, ground truth the best, and our reconstructions consistently outperformed PC2. These results confirm that the regulators can reliably distinguish point clouds by type and model, and that their supervision provides meaningful priors that effectively enhance the performance of our diffusion model.

## 6. Conclusions

In this work, we tackled the challenging problem of reconstructing fine-grained 3D vehicle point clouds from a single non-ideal image, where severe viewpoint limitations, occlusions, and background clutter lead to highly ambiguous geometry. Our main contribution is a prior-guided conditional diffusion framework that injects rich geometric and semantic priors into both the denoising process and the training objective. Concretely, we (i) fused camera-aware image features, masks, and distance-transform maps with the evolving point cloud to provide strong geometric control; and (ii) embedded textual prompts of the form “Brand; Model; Type” through cross-attention, enabling vehicle-specific semantic guidance. On top of this, we introduced frozen Vehicle Type and Vehicle Model Regulators as semantic regularizers that enforce consistency with learned type distributions and text–geometry alignments. Extensive experiments on the 3DRealCar++ dataset show that our method significantly improves over state-of-the-art single-view point cloud reconstruction baselines.

In practical deployment scenarios, the proposed framework is primarily designed for offline or semi-online 3D asset generation in autonomous driving simulation and digital-twin systems, rather than strict real-time onboard perception. Therefore, reconstruction fidelity and semantic consistency are prioritized over ultra-low-latency inference. Nevertheless, the DDIM-based sampling strategy provides substantially faster inference while maintaining competitive reconstruction quality, demonstrating a favorable trade-off between computational efficiency and reconstruction performance.

Although the proposed framework utilizes semantic prior information such as vehicle brand, model, and category attributes, the objective is not to memorize fixed vehicle templates but to learn generalized semantic–geometric correlations across vehicle structures. Therefore, the proposed framework can maintain a certain generalization capability toward unseen or newly introduced vehicle variants. In practical deployment scenarios, newly emerging vehicle models can be incorporated through incremental dataset expansion and lightweight fine-tuning without modifying the overall reconstruction architecture. The text-prompt-based semantic guidance mechanism also provides a flexible way to extend the semantic space as new vehicle categories and models become available.

Future research may further improve the practicality of the proposed framework by reducing the dependence on human intervention during semantic prior generation. In particular, integrating vision–language models and automatic vehicle recognition systems may enable semantic priors such as vehicle brand, model, and type to be extracted directly from unconstrained real-world images. In addition, continual learning strategies could help the reconstruction model incrementally adapt to newly emerging vehicle models without requiring complete retraining. Exploring self-supervised semantic alignment mechanisms that jointly learn geometric and semantic consistency without explicit human verification also represents a promising direction toward fully automated and scalable vehicle reconstruction systems.

## Figures and Tables

**Table 1:** Methodological comparison between the proposed method and representative single-view 3D reconstruction approaches.

Method	Representation	Diffusion-Based	Semantic Prior	Text Guidance	Non-Ideal View Robustness	Semantic Regularization
RGB2Point	Point Cloud	×	×	×	Weak	×
PC2	Point Cloud	✓	×	×	Moderate	×
BDM	Point Cloud	✓	Partial	×	Moderate	×
PixelNeRF	NeRF	×	×	×	Weak	×
One-2-3-45++	✓	×	×	Moderate	×	
Spar3D	Mesh	✓	×	×	Moderate	×
VQA-Diff	Multi-View Images	Partial	✓	✓	Moderate	×
Ours	Point Cloud	✓	✓	✓	Strong	✓

**Table 2:** Prior-guided conditional denoising pipeline for a single step of $S_{\theta }\left(X_{t-1}|X_{t},C\right)$.

**Input**	Noisy point cloud $X_{t}\in R^{3\times N}$, image $I\in R^{H\times W\times 3}$, camera intrinsics **K**, extrinsics **T**, text prompt *T*.
**Step 1**	Obtain mask $M\in R^{H\times W}$ from *I* using LSAM [45]. Compute distance map $D\in R^{H\times W}$ by $D\left(p\right)=\mathrm{Norm}\left(\min_{q\in V}{∥p-q∥}_{2}\right)$, where $V=\{\left(x_{q},y_{q}\right)|M\left(x_{q},y_{q}\right)=1\}$. Extract image features $F_{\mathrm{rgb}}\in R^{H\times W\times F_{1}}$ with ViT and form $F_{2D}=\mathrm{Concat}\left(I,M,D,F_{\mathrm{rgb}}\right)\in R^{H\times W\times (3+1+1+F_{1})}$.
**Step 2**	Project $F_{2D}$ onto $X_{t}$ using rasterizing projection with (K,T) to obtain $F_{3D}=P_{r}\left(X_{t},F_{2D},K,T\right)\in R^{(5+F_{1})\times N}$. Augment the noisy point cloud as $X_{\mathrm{aug}}=\mathrm{Concat}\left(F_{3D},X_{t}\right)\in R^{(5+F_{1}+3)\times N}$.
**Step 3**	Encode text prompt *T* using CLIP [19] to get $F_{\mathrm{text}}\in R^{F_{2}}$.
**Step 4**	Fuse $X_{\mathrm{aug}}$ and $F_{\mathrm{text}}$ via fully connected layers and a cross-attention module, yielding $X_{+}=\mathrm{CrossAtten}\left(X_{\mathrm{aug}},F_{\mathrm{text}}\right)\in R^{(5+F_{1}+3)\times N}$.
**Step 5**	Use $X_{+}$ as the condition of the diffusion model to predict noise $\epsilon_{\mathrm{pred}}=S_{\theta }\left(X_{0}|X_{t},C\right)=S_{\theta }\left(X_{0}|X_{+}\right)$, and update the sample with a DDPM/DDIM-style rule $X_{t-1}=\mathrm{UpdateStep}\left(X_{t},\epsilon_{\mathrm{pred}},t\right)$.

**Table 3:** Category-wise image distribution of the 3DRealCar++ dataset.

Categories	Images
Sedan	4310
SUV	5658
Van	2235
Pickup	2091
Sportcar	3398
MPV	2987
Others	3210
**Total**	**23,889**

**Table 4:** Quantitative comparison with existing methods on the 3DRealCar++ dataset.

Method	Prec. ↑	Rec. ↑	F1-Score ↑	CD ↓
RGB2Point [27]	0.3411	0.6108	0.4157	89.8521
SDFit [55]	0.5523	0.6417	0.5742	84.3765
ICDDPM [56]	0.6184	0.6592	0.6157	78.5349
PC2 [29]	0.6071	0.6196	0.5938	81.7665
BDM [32]	0.6280	0.6439	0.6163	79.2421
Ours	**0.7020**	**0.7278**	**0.6990**	**70.0585**
Ours-DDPM	**0.7399**	**0.7395**	**0.7226**	**62.1525**

**Table 5:** Per-category F-score comparison on the 3DRealCar++ dataset.

Category	RGB2Point [27]	SDFit [55]	ICDDPM [56]	PC2 [28]	BDM [29]	Ours
Sedan	0.4305	0.6016	0.6629	0.6219	0.6668	0.7441
SUV	0.4085	0.5932	0.6592	0.6138	0.6960	0.7364
Van	0.3779	0.5357	0.5410	0.5331	0.5487	0.6320
Pickup	0.4268	0.5830	0.6133	0.6121	0.6110	0.7048
Sport Car	0.3580	0.5598	0.6035	0.5514	0.6005	0.6649
MPV	0.4827	0.5904	0.6471	0.6081	0.6489	0.7184
Others	0.4258	0.5559	0.5827	0.6160	0.5716	0.6924
**Avg. F-score ↑**	0.4157	0.5742	0.6157	0.5938	0.6163	**0.6990**

**Table 6:** Ablation study on the impact of regulators.

$L_{\mathrm{VTR}}$	$L_{\mathrm{VMR}}$	Prec. ↑	Rec. ↑	F1-Score ↑	CD ↓
**✗**	**✗**	0.6440	0.6577	0.6371	77.4323
**✓**	**✗**	0.6716	0.7062	0.6894	71.7480
**✗**	**✓**	0.6603	0.6850	0.6807	74.4547
**✓**	**✓**	**0.7020**	**0.7278**	**0.6990**	**70.0585**

**Table 7:** Ablation study on the fusion methods.

Experiment	Prec. ↑	Rec. ↑	F1-Score ↑	CD ↓
No text prior (PC2)	0.6071	0.6196	0.5938	81.7665
Broad fusion	0.6343	0.6390	0.6274	77.5601
**Attention fusion**	**0.6440**	**0.6577**	**0.6371**	**77.4323**

**Table 8:** Ablation study on different text encoders for semantic priors.

Text Encoder	Prec. ↑	Rec. ↑	F1-Score ↑	CD ↓
No text prior	0.5938	0.6012	0.5824	83.4173
BERT [57]	0.6815	0.7037	0.6719	72.8564
RoBERTa [58]	0.6927	0.7159	0.6858	71.1925
**CLIP (ours)**	**0.7020**	**0.7278**	**0.6990**	**70.0585**

**Table 9:** Ablation study on different diffusion noise schedules (DDIM, 100 steps).

Noise Schedule	Prec. ↑	Rec. ↑	F1-Score ↑	CD ↓
Cosine	0.6932	0.7185	0.6951	71.4827
Quadratic	0.6864	0.7093	0.6884	72.9062
**Linear (ours)**	**0.7020**	**0.7278**	**0.6990**	**70.0585**

**Table 10:** Ablation study on different training schemes for the regulators.

Training Scheme	Prec. ↑	Rec. ↑	F1-Score ↑	CD ↓
W/o regulators	0.6440	0.6577	0.6371	77.4323
Joint training (unfrozen)	0.6842	0.7101	0.6923	72.9132
**Pretrained + frozen (ours)**	**0.7020**	**0.7278**	**0.6990**	**70.0585**

**Table 11:** Ablation study on sampling strategies and sampling steps.

Exp.	Prec. ↑	Rec. ↑	F1-Score ↑	CD ↓	Infer. Time ↓
ddpm-100 steps	0.6833	0.7225	0.6929	73.3210	~2.7 s
ddim-100 steps	0.7020	0.7278	0.6990	70.0585	~2.6 s
**ddpm-1000 steps**	**0.7400**	**0.7395**	**0.7226**	**62.1525**	**~26.7 s**

**Table 12:** Ablation study on the necessity and effectiveness of the regulators.

Method	Instance Accuracy ↑	Matching Score ↓
Ground Truth	0.9536	3.7391
PC2	0.4119	4.8035
Ours	0.5778	4.1256
Noise	0.0121	23.8367

## Data Availability

All data covered in this paper are available from the corresponding author upon request.
